# Phosphoinositide 3-Kinases as Potential Targets for Thrombosis Prevention

**DOI:** 10.3390/ijms23094840

**Published:** 2022-04-27

**Authors:** Natasha M. Setiabakti, Pia Larsson, Justin R. Hamilton

**Affiliations:** Australian Centre for Blood Diseases, Monash University, Melbourne, VIC 3004, Australia; natasha.setiabakti@monash.edu (N.M.S.); pia.larsson@monash.edu (P.L.)

**Keywords:** platelets, thrombosis, phosphoinositide 3-kinase, PI3K, antiplatelet therapy

## Abstract

As integral parts of pathological arterial thrombi, platelets are the targets of pharmacological regimens designed to treat and prevent thrombosis. A detailed understanding of platelet biology and function is thus key to design treatments that prevent thrombotic cardiovascular disease without significant disruption of the haemostatic balance. Phosphoinositide 3-kinases (PI3Ks) are a group of lipid kinases critical to various aspects of platelet biology. There are eight PI3K isoforms, grouped into three classes. Our understanding of PI3K biology has recently progressed with the targeting of specific isoforms emerging as an attractive therapeutic strategy in various human diseases, including for thrombosis. This review will focus on the role of PI3K subtypes in platelet function and subsequent thrombus formation. Understanding the mechanisms by which platelet function is regulated by the various PI3Ks edges us closer toward targeting specific PI3K isoforms for anti-thrombotic therapy.

## 1. Introduction

Cardiovascular disease is the leading cause of death across the globe, accounting for 31% of all deaths worldwide [1]. Most cardiovascular diseases are caused by pathological arterial thrombosis formed following an atherosclerotic plaque rupture or vessel injury. These thrombi are composed predominantly of aggregates of activated platelets and fibrin. A detailed understanding of platelet function in settings of haemostasis and thrombosis is thus key to design safe and effective treatments that prevent thrombotic cardiovascular disease without significant disruption of the haemostatic balance.

Platelet activities and functions during thrombus formation have been extensively reviewed elsewhere [2]. In brief, when vessel injury occurs, various thrombogenic subendothelial matrix proteins are exposed. Circulating platelets have the ability to use their array of surface adhesion receptors to rapidly stick to these exposed subendothelial matrix components (primary platelet adhesion) [2,3]. Platelets express various integrin receptors including α_2_β_1_ and α_IIb_β_3_ that bind to adhesive proteins [4,5]. Binding of these integrins to their respective ligands, in combination with activation of non-integrin surface receptors including GPVI, then initiates intracellular signalling pathways to stabilise platelet adhesion and trigger platelet recruitment and aggregation [2,6]. By generating and releasing various soluble agonists including ADP, thrombin, and thromboxane A2 (TxA2), adhered and activated platelets propagate the stimulatory signal away from the vessel wall to recruit more free-flowing platelets to the growing thrombus mass (platelet aggregation). Activation of integrin αIIbβ_3_ and subsequent fibrinogen-mediated platelet-platelet bridging and outside-in signalling provides a support for platelet aggregation [7,8]. A subset of highly activated platelets also form a scaffold for fibrin formation due to their ability to support thrombin activation on their surface [9]. Altogether, these activities of platelets make up the basis of clot formation. A shift in any of these activities can thus disrupt the delicate balance of haemostasis, leading to either bleeding or thrombosis.

Strategies for the management and prevention of arterial thrombosis are largely based on pharmacological modulation of platelet activation and aggregation via inhibition of the soluble agonists outlined above. The clinical benefit of using anti-platelet agents in the prevention of cardiovascular diseases has been long established especially in the setting of secondary prevention. The three most common clinically used classes of anti-platelet agents, i.e., cyclooxygenase-1 inhibitor (aspirin), ADP signalling (P2Y12) inhibitors (clopidogrel, ticagrelor, or prasugrel), and GPIIb/IIIa inhibitors, all target platelet signalling pathways that prevent global platelet activation [10,11]. Dual anti-platelet therapy (DAPT), the combination of aspirin with a P2Y12 receptor inhibitor, inhibits platelet activation by blocking both the production of thromboxane and the ADP signalling pathway. This aggressive treatment strategy consistently reduces the relative risk of cardiovascular events but at the expense of bleeding complications [12,13]. Despite the bleeding risk, DAPT is still the standard of care for the prevention of recurrent cardiovascular diseases [14,15,16] and cerebrovascular diseases [11].

Further, currently used anti-platelet therapeutic strategies are less effective in certain large and important patient groups, including in patients with metabolic disease [17,18,19]. There is some evidence that metabolic syndrome can cause antiplatelet resistance [20]. The prevalence of aspirin resistance has been reported as 24% of all individuals [21], including in various patient groups such as the elderly [22] and in those with metabolic diseases [23,24]. Furthermore, the OPTIMUS (Optimizing Antiplatelet Therapy in Diabetes Mellitus) trial highlighted that even when the typical clinical dose of clopidogrel was doubled, nearly 60% of diabetes patients still have a poor response to this antiplatelet agent [25]. These and other factors result in sub-optimal thrombosis prevention in large and growing patient cohorts. Thus, there is a need for novel anti-thrombotic targets to be developed.

Ideally, an antiplatelet therapy should inhibit pathological thrombus formation while largely preserving the haemostatic function of platelets. By targeting global platelet activation, via inhibition of TxA2 and ADP, current treatments do not effectively discriminate between attenuating platelet function in the setting of haemostasis and thrombosis. Indeed, distinguishing the two major platelet functions remains a major challenge in the field. A recently emerging prospective anti-thrombotic target that could prove to be more selective towards thrombosis, and possibly effective in a wider range of patients, is the phosphoinositide 3-kinase (PI3K) lipid kinase family. PI3K phosphorylates the inositol ring of phosphatidylinositol (PI) to generate a secondary lipid signalling for cell functioning [26]. The PI3K family is involved in various aspects of platelet biology and function, and therefore could be a promising candidate for a more targeted anti-platelet agent. This review will describe the PI3K family members with the focus on their role in platelet biology and thrombus formation, and their potential strengths and challenges for the use as anti-thrombotic agents.

## 2. Phosphoinositide 3-Kinase

Phosphoinositide 3-kinase (PI3K)/Akt/mTOR is a crucial intracellular signalling pathway that regulates various cellular events such as cell growth, metabolism, and vesicular trafficking. PI3Ks phosphorylate the 3′ position of the hydroxyl group in the inositol group of the PI resulting in 3-phosphoinositides (3-PPI). There are seven phosphorylated derivatives of PI in cells depending on the position and the number of the phosphorylated hydroxyl group; PI-3-phosphate (PI(3)P), PI-4-phosphate (PI(4)P), PI-5-phosphate (PI(5)P), PI-3,4-biphosphate (PI(3,4)P_2_), PI-3,5-biphosphate (PI(3,5)P_2_), PI-4,5-biphosphate (PI(4,5)P_2_), and PI-3,4,5-triphosphate (PI(3,4,5)P_3_), with the PI3Ks responsible for generating each of the species phosphorylated at the 3′ position [26,27].

There are three broad groups of PI3K enzymes: Class I, Class II, and Class III. All PI3Ks consist of a PI3K core (catalytic domain, helical domain, and a C2 domain) and a variety of additional subunits including regulatory subunits [28]. The difference between the three classes of PI3Ks lies on additional subunits attached to it as well as on its lipid substrate. For instance, Class I PI3K function together with a regulatory subunit attached [29], while Class II PI3Ks have an amino- and carboxy-extension to the PI3K core subunit, and the single Class III PI3K, vps34, lacks a regulatory subunit [28,30].

Most of the work investigating the function of PI3K and their 3-PPI products has focused on their involvement in cell signalling, including intracellular signalling required for platelet activation [31]. Amongst all the three classes of PI3Ks, the Class I are the most widely studied isoforms, with recently emerging studies throughout other classes. In the last decades, inhibitors against PI3Ks have been developed and investigated as a potential novel pharmacological treatment [32].

## 3. Class I PI3K

Class I PI3Ks all contain a catalytic subunit, called p110, and a regulatory subunit that maintains the activity of the enzyme [33]. There are four distinct Class I PI3K isoforms: PI3Kα, PI3Kβ, PI3Kδ, and PI3Kγ. Class I PI3Ks generate their main product, PI(3,4,5)P_3_, by 3-phosphorylating PI(4,5)P_2_. This lipid product is important to regulate cell survival, proliferation [34], and migration, as well as glucose transport [35]. PI3Kα and PI3Kβ are widely expressed in mammalian cells, while PI3Kδ and PI3Kγ expression is more restricted, and is found predominantly in haematopoietic cells [36,37]. Extensive studies have been performed on Class I PI3Ks and their relation with human diseases. All Class I PI3K isoforms have been implicated in cell proliferation and cancer [38,39,40,41,42,43]. PI3Kδ and PI3Kγ have been described to have a role in the immune system [44,45,46] such as anaphylactic reaction [47] and inflammatory disease reaction including systemic lupus erythematosus [48] and rheumatoid arthritis [49]. The importance of Class I PI3K in regulating cell survival has led to the approval of several Class I pharmacological inhibitors to be used in various cancer treatments [50].

### Role of Class I PI3Ks in Platelet Function and Thrombus Formation

Human platelets express all Class I PI3K isoforms [51]. Using selective pharmacological inhibitors and mouse models, Class I PI3K has been shown to have an important function in platelet signalling and function [52,53,54,55,56].

It was initially observed that PI3Kα-deficient mice had an impaired platelet aggregation in response to low concentrations of the GPVI agonists, collagen, and collagen-related peptide (CRP) [57]. A similar but exaggerated response was seen when human blood was pretreated with a pharmacological inhibitor of PI3Kα, PIK75, resulting in complete inhibition of the aggregation response induced by CRP, collagen, and thrombin [58]. Genetic deletion of PI3Kα resulted in normal and stable thrombus formation in ex vivo thrombosis flow experiments [55,56]. Additionally, PI3Kα-deficiency decreased thrombus formation following a laser-induced vessel injury with no significant tail bleeding prolongation [57].

Of the four Class I isoforms, PI3Kβ appears to have the highest level of expression [59] and a major role in platelet activation mechanisms. Platelets form and accumulate PI(4,5)P_2_ and PI(3,4,5)P_3_ for cell signalling and regulation of platelet adhesion in response to thrombin [60]. A mouse model with conditional deletion of PI3Kβ in platelets showed that PI3Kβ is crucial for PI(3,4,5)P_3_ production in response to various agonists. This finding was supported by studies with a PI3Kβ specific inhibitor (TGX-221) which also showed strongly impaired PI(3,4,5)P_3_ production following exposure to platelet agonists [52].

Platelets from PI3Kβ-deficient mice consistently exhibit impaired responses in both in vitro platelet aggregation assays and ex vivo platelet function assays (i.e., whole blood thrombosis assays) [52,55]. Furthermore, following ferric chloride carotid artery injury, PI3Kβ-deficient mice were protected from complete occlusion of the vessel, with no prolonged bleeding time, indicating prevention of thrombosis with preserved haemostatic function [52]. However, administration of the PI3Kβ inhibitor, TGX-221, at an anti-thrombotic dose resulted in prolongation of tail and kidney bleeding time in mice [61]. Following these early studies of PI3Kβ in platelet function, small molecules specifically targeting PI3Kβ have been developed, including AZD6482 [54]. Treating human blood with AZD6482 inhibits platelet aggregation. This anti-platelet effect was most obvious in the presence of blood flow [54]. AZD6482 administration in dogs resulted in no significant increase in bleeding time and an anti-thrombotic effect in the Folt’s model of thrombosis [54]. Intravenous infusion to healthy male volunteers replicated the inhibition of platelet aggregation but with a 60% increase in the cutaneous bleeding time at the highest plasma concentration [54].

As current standard-of-care often involves a combination of two antiplatelet agents, the study extended to look at the efficacy and bleeding risk in AZD6482 monotherapy and combined therapy with either aspirin or P2Y12 antagonists (clopidogrel) in dogs and healthy male volunteers [62]. The combination of aspirin and AZD6482 caused a more pronounced inhibition of platelet aggregation and less cutaneous bleeding than that observed with the combination of aspirin and clopidogrel [62].

PI3Kδ is expressed in both human and mouse platelets [63]. Mice with PI3Kδ-deficiency were shown to have a partial reduction in platelet aggregation when stimulated with a low concentration of CRP. This result was replicated with human blood pretreated with the PI3Kδ inhibitor, Idealisib [58]. Despite this, genetic deletion of PI3Kδ in mice does not seem to affect thrombus formation [63]. Yet pharmacological inhibition of PI3Kδ in mice resulted in delayed occlusive thrombus formation in the ferric chloride-induced injury model and prolonged tail bleeding time [58].

The PI3Kγ isoform has also been shown to have a role in platelet function. Mice deficient in PI3Kγ have impaired platelet aggregation in response to ADP and are protected against thromboembolism [64]. PI3Kγ-deficient mice also showed unstable thrombus formation both in a carotid artery in vivo thrombosis model [65] and when perfused over a collagen/vWF surface in ex vivo blood flow experiments [66]. Together with PI3Kβ, PI3Kγ appears responsible for integrin α_IIb_β_3_ activation and subsequent platelet aggregation [67]. 

## 4. Class II PI3K

There are three known isoforms of Class II PI3Ks: PI3KC2α, PI3KC2β, and PI3KC2γ. PI3KC2α and PI3KC2β are widely expressed in humans [68], including in platelets [69]. In contrast, PI3KC2γ expression is limited to the liver, breast, prostate, and salivary gland [70], with no platelet expression [69]. Class II PI3Ks have overlapping capability to produce PI(3)P and PI(3,4)P_2_ [71,72] which are involved in vesicular trafficking, glucose regulation, endocytosis, cell growth, and other intracellular signalling [71]. 

Emerging studies regarding the cellular functions of PI3KC2α shows that this isoform plays a major role in cell metabolic activities, growth, and survival [70,73,74,75].The first known human monogenic disorder caused by loss of function mutation of PI3KC2α was reported recently [76]. Three unrelated consanguineous families presented with a novel syndrome involving growth retardation and skeletal, neurological, visual, and hearing abnormalities. In contrast to the viability in humans, genetic deletion of the PI3KC2α gene in mice leads to impaired embryonic development from gestational age 8.5 and fatality at e10.5-11.5 due to impaired angiogenesis [77]. PI3KC2α is highly expressed in endothelial vascular tissues and deficiency of this enzyme leads to defective vascular formation and barrier integrity [77]. It was reported that PI3KC2α deficiency showed a decreased level of basal pool of PI(3)P and hence disrupted cilia elongation [76,78]. Whether the cause of the discrepancy in effects caused by PI3KC2α-deficiency in humans and mice is the result of functional differences in the enzyme, compensation by PI3KC2β (or other protein/s) function, or some other mechanism remains unknown. Regardless, PI3KC2α plays a crucial role in normal development in both humans and mice.

Contrary to the lethality observed with PI3KC2α-deficient mice, PI3KC2β knockout mice are viable and show normal development and growth [71]. PI3KC2β is widely expressed both in human and mouse tissues, with a known role in cancer cell migration and motility. Inactivation of this enzyme also showed a role in insulin signalling and glucose homeostasis [79,80]. A recent study showed that inhibiting PI3KC2β reduces cerebral infarct and improves neurological outcomes [81]. This is thought to be by preserving vascular integrity that resulted in stabilisation of the blood–brain barrier following ischemic events and reduces brain inflammation [81].

### Role of Class II PI3K in Platelet Function and Thrombus Formation

The role of Class II PI3Ks in platelets has just started to emerge. Both PI3KC2α and PI3KC2β, but not PI3KC2γ, are expressed in platelets [82]. Despite the finding that PI3KC2β appears to be expressed at higher levels than PI3KC2α in platelets, a PI3KC2β-deficient mouse exhibited normal platelet function, haemostatic response, and no difference in the level of the basal or agonist-stimulated level of PI(3)P in platelets [83].

Given that global and constitutive genetic deficiency of PI3KC2α is embryonically lethal in mice, two different mouse models were used to generate adult mice with PI3KC2α-deficiency, one using an inducible shRNA-based approach and one a heterozygous point mutation causing a kinase-dead version of the enzyme [83,84]. In both of these mouse models, it was observed that PI3KC2α is essential for platelet membrane structure and overall function. Platelets have a unique internal membrane reserve system, known as the open canalicular system (OCS). The OCS is a membrane surface that folds inward and is critical for platelet activation [85]. This surface-connected membrane reserve has several functions; it provides the increased platelet surface area required for the formation of filopodia that occur when a platelet becomes activated, it acts as a storage unit for platelet glycoprotein (GP) complexes, and it functions as an exchange pathway for molecules to travel into and out of the cell. Using an inducible shRNA approach that resulted in global deficiency of PI3KC2α and a near-deletion in platelets, it was found that the OCS in platelets is enlarged [83]. Other than this increased surface area of OCS, there were no other differences reported in OCS distribution or ultrastructure between platelets isolated from PI3KC2α-deficient or littermate controls [83]. Further observation found that despite the mild membrane defect, PI3KC2α deficiency did not have any impact on the lipid composition of the platelet membrane [86]. This finding was then independently confirmed by a separate research group that observed the same platelet defect in mice that were heterozygous for a point mutation that causes inactivation of the enzymatic activity of PI3KC2α [84]. In addition, these authors also demonstrated rigidification of the platelet plasma membrane and mislocation of cytoskeletal proteins with an increase in barbell-shaped proplatelets [84]. It was also found that there were no differences in the activated level of phosphorylated PI, but rather a reduction in the basal pool of PI(3)P in platelets from PI3KC2α-deficient mice [84,87].

Additionally to these in vitro observations regarding platelet structure and function, PI3KC2α-deficient mice were protected against thrombosis, with any thrombi that formed being both delayed and unstable following an electrolytic-injury model of in vivo thrombosis [83]. Despite this protection against thrombosis, PI3KC2α-deficiency resulted in preserved haemostatic function, characterised by a normal tail bleeding time. Further studies looking at the combined deficiency of PI3KC2α and PI3KC2β showed that impaired thrombus formation and enlarged OCS are specific to PI3KC2α deficiency and not enhanced when both enzymes are missing from mouse platelets [88].

One of the challenges in translating these findings in mice to any human relevance has been the lack of specific pharmacological inhibitors of PI3KC2α. Recently though, the first generation of pharmacological PI3KC2α inhibitors with limited off-target effects towards other PI3K isoforms were developed—MIPS-19416 and MIPS-21335 [87]. Treating human and mouse platelets with MIPS-19416 reproduced the OCS dilation to a similar extent as that observed in platelets from PI3KC2α-deficient mice [87]. In a series of experiments using a range of pharmacological agents to control for any off-target effects on other PI3K isoforms, this effect appeared highly specific to PI3KC2α inhibition [87]. Marked OCS dilation caused by PI3KC2α inhibition happened as quickly as 5 min after drug treatment and was fully reversible after drug washout, suggesting a highly dynamic membrane structure [87]. In a further replication of the findings in PI3KC2α-deficient mice, treatment of human platelets with PI3KC2α inhibitors reproduced the observations of a reduction in basal PI(3)P level [87]. Consistently, the reduction in PI(3)P level was not observed in activated platelets. This once again support the previous findings that PI3KC2α is responsible for maintaining a basal pool of PI(3)P. Finally, pharmacological inhibition of PI3KC2α in human blood caused an antithrombotic effect in an ex vivo thrombosis model comparable to the effects of conventional antiplatelet agents (aspirin and P2Y12 antagonist). A similar antithrombotic effect of PI3KC2α inhibitor was also observed in experiments performed at higher shear rate in which the conventional antiplatelet agent effect has diminished [87]. Occlusive thrombus formation in the in vivo mouse model following electrolytic injury was also significantly reduced after intravenous pre-treatment of PI3KC2α inhibitor. Similar to the PI3KC2α knockdown mouse model [83], pharmacological inhibition of PI3KC2α does not show prolonged tail bleeding time and blood loss. All in all, pharmacological inhibition of PI3KC2α in human blood and isolated platelets reproduced almost all of the effects observed in several distinct models of genetic PI3KC2α-deficiency.

## 5. Class III PI3K

The single Class III PI3K is known as vacuolar protein-sorting defective 34 (Vps34) [89] that exclusively generates stimulation-dependent PI(3)P. Vps34 has been associated with a critical role in autophagy, endocytosis, and mTOR signalling [90]. It has been reported that the total knockout of the enzyme is embryonically lethal [91,92]. Hence, to overcome this, most Class III PI3K studies had been undertaken by using a conditional knockout in different tissues using the Cre-Lox system. Vps34-deficient liver mouse models are found to later develop hepatomegaly and hepatosteatosis, whereas Vps34-deficient heart mouse models present weaker myocardial contractility and cardiomegaly; which cause both models to die as early as eight weeks of age [90]. Until recently, there was only one human disease-related Vps34 expression reported, a correlation between Vps34 mutation with bipolar disorder and schizophrenia [93,94]. However, it has now been reported that Vps34 deletion in kidney proximal tubules cause renal failure [95].

### Role of Class III PI3K in Platelet Function and Thrombus Formation

Only in recent years it has been reported that Vps34 has a role in platelet activation. Two independent groups have developed megakaryocyte/platelet-specific Vps34 deficient mouse lines, both of which were viable with normal hematologic parameters as well as platelet ultrastructure and protein expression level [96,97]. However, one group found that the Vps34-deficient mice have mild microthrombocytopenia due to impaired platelet production with reduced alpha- and dense granules [96]. This is further explained by examining the effect of localisation of PI(3)P at platelet precursor cells, megakaryocytes [98]. Megakaryocyte maturation, and consequently proplatelet formation, is highly regulated by Vps34-derived PI(3)P. Inhibiting Vps34 in immature megakaryocytes showed a reduction in the demarcation membrane system (DMS) as well as in formation of proplatelets, which are solely due to impaired vesicle trafficking [98]. 

Despite the mild abnormality in platelet number and morphology observed in Vps34-deficient mice, these mice did exhibit impaired platelet function and thrombus formation [96,97]. Reduced platelet function in Vps34^−/−^ platelets was confirmed by impaired platelet aggregation, integrin activation, and granule secretion in response to low concentrations of various agonists [96,97]. Consequently, there was a delayed thrombotic response in a ferric chloride-induced mesentery artery injury model of in vivo thrombosis, as well as thrombus formation ex vivo under arterial flow in Vps34-deficient mice [96,97]. More importantly, a similar thrombus growth defect was also observed in human blood treated with two unrelated Vps34-specific inhibitors [96]. Similar to the other classes of PI3K, Vps34-deficient mice showed normal haemostatic function of platelets reflected by tail bleeding time [96,97].

Understanding the mechanism behind this continues to be the challenge in the area. Vps34-deficiency does not show impaired platelet spreading on immobilised fibrinogen indicating a normal early stage outside-in signalling pathway, but a significantly delayed clot retraction [97]. Consistent with the function of Class III PI3K, the effect of Vps34 deficiency has started from the precursor megakaryocytes cells [98]. Furthermore, stimulated PI(3)P production was lower in Vps34-deficient platelets, with a comparable basal PI(3)P level [96,97]. It was also reported that Vps34 inactivity resulted in accelerated platelet granule secretion that leads to defective platelet recruitment in thrombus under arterial shear [96]. Another potential mechanism that has been suggested is that Vps34 inactivation leads to decreased NADPH oxidase-dependent ROS generation and consequent mTOR signalling [97].

## 6. PI3Ks as Anti-Thrombotic Targets?

The extent and use of antiplatelet therapy in preventing cardiovascular events has been long debated, both in primary and secondary prevention. The use of antiplatelet agents in primary prevention is now rarely recommended due to its questionable benefits with the cost of increased bleeding risk [99]. Long-term use of aspirin for primary prevention of cardiovascular events significantly increases risk of major bleeding without lowering the risk of cardiovascular events in elderly [100]. This has resulted in limitation of the use of antiplatelet therapy, especially in patients at a high risk of bleeding.

**Figure 1:**
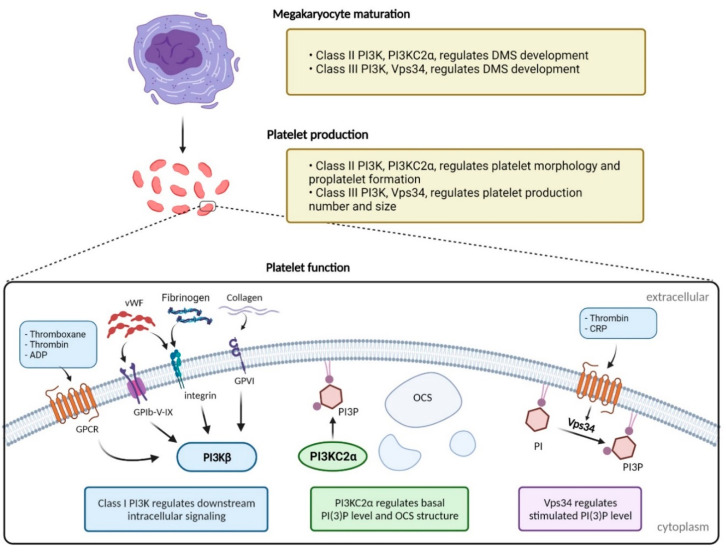
The function of PI3K isoforms in megakaryocytes and platelets. The sole Class III PI3K, Vps34, plays important roles in both platelet production and function while specific Class I and Class II isoforms are more selectively involved in platelet function. The Class I PI3K, PI3Kβ, is involved in intracellular signalling downstream of many platelet surface receptors including GPCRs and integrins and contributes to the platelet activation mechanisms that support thrombus growth and stabilisation. The Class III isoform, Vps34, appears largely responsible for forming PI3P (and its subsequent intracellular signalling events) in response to GPCR activation. In contrast, the Class II isoform, PI3KC2α, does not phosphorylate PI into PI3P in response to agonist activation but rather maintains basal PI3P levels and regulates the internal cell membrane (OCS) structure. OCS = open canalicular system; DMS = demarcation membrane system; GPCR = G protein coupled receptor; vWF = von Willebrand factor; CRP = collagen-related peptide. Figure created with BioRender.com.

There has been a need to develop novel antiplatelet therapies that are effective in preventing thrombosis but that do not inhibit global platelet activation. As summarised in this review, all three classes of PI3K play a role in platelet function through different mechanisms (see Table 1 and Figure 1) and could potentially be attractive new targets for pharmacological intervention that meets these criteria. Most of the spotlight so far has fallen on Class I PI3Ks, and the p110β isoform in particular. It is clear that p110β has a major role in platelet function and thrombus formation, and thus received the most attention as a potential anti-thrombotic target. This has led to the clinical development of the first PI3K inhibitor in the thrombosis field. The small molecule PI3Kβ inhibitor, AZD6482 [54,62], has entered clinical trials and showed promising efficacy and safety profile in pre-clinical animal models and phase I safety trials in humans, both alone and in combination with standard-of-care antiplatelet therapy (aspirin) [62]. Although promising, one study showed that PI3Kβ is responsible for thrombus dynamics and stability at a pathological shear rate in both mice and humans [55]. As thrombus consolidation is affected by α_IIb_β_3_ outside-in signalling [101] and hence dependent on the PI3Kβ signalling pathway, targeting PI3Kβ may lead to thrombus porosity and instability at a higher shear rate. The thrombus instability and embolism potential are seen as a risk for secondary thromboembolic events [52,55,102]. Another downside was the short half-life and rapid onset of action of AZD6482 [54] which means that a continuous infusion might be required to sustain platelet inhibition. Currently, the progression of the clinical programs have been halted, although whether or not this is due to increased thrombus instability and risk of embolization as seen in animals models [52] or some other reason/s is unknown.

As the clinical development of the PI3Kβ inhibitor for thrombosis has halted, it opens the door for the potential use of Class II and III PI3K inhibitors. Deficiency in Class I and III PI3K isoforms leads to measurable defects in in vitro platelet functional response to various agonist, as seen in aggregation and activation assays [52,55,63,64,66,97]. This might indicate that targeting these classes might still not be able to fully discriminate thrombosis from haemostasis, as has been observed with the bleeding tendencies after the use of PI3Kβ inhibitor, TGX-221 [61] and mild bleeding defect in the AZD6482 clinical trial [54]. In contrast, targeting the Class II PI3K, PI3KC2α, either genetically or pharmacologically, appears to have no measurable effects on readouts of global platelet function measured by platelet aggregation, integrin activation, and granule release [83,87]. The limited mechanistic studies available so far suggest that the anti-thrombotic effect associated with genetic or pharmacological PI3KC2α deficiency is specific to shear-dependent platelet–platelet interaction [83,84,87]. It is likely that the impaired thrombus formation seen in PI3KC2α deficiency is correlated with its function to maintain basal PI(3)P levels and its role in regulating platelet internal membrane (OCS) [83,86], rather than modulating intracellular signalling as seen for Class I PI3K and also with traditional antiplatelet therapy. In contrast, deficiency in the other isoform of Class II PI3K, PI3KC2β does not impact thrombus formation and thus does not appear as an attractive target for thrombus prevention [83].

The finding that PI3KC2α is only affecting platelet adhesion in a shear-specific way, with preserved global platelet function, makes it an intriguing target. However, the critical role of the PI3KC2α enzyme in growth and development as evidenced by the embryonic lethality of PI3KC2α deficient mice and a novel syndromic growth in human loss-of-function of PI3KC2α leads to the key question about possible adverse effects of long-term treatment and a potentially narrow therapeutic window.

## 7. Future Directions

For any new anti-platelet agent under development, the two major limitations associated with current anti-platelet therapy, i.e., the increased bleeding risk and reduced efficacy in certain high-risk categories of patients, need to be considered. Although it has been consistently shown that targeting one or more isoforms of PI3K does not prolong tail bleeding times in mice, this needs to be confirmed in other animal models of haemostasis as well as in human clinical trials. Regarding the problem of limited efficacy in certain patient groups, any novel anti-thrombotic agent should be tested in high-risk patient groups where current antiplatelet therapy has served poorly. It has been reported that a daily administration of aspirin only reduced platelet reactivity by 14.1% in diabetic patients, compared to 78.6% in the general population [103] making diabetic patients an interesting group to examine. However, considering the effect of PI3K enzymes in regulating metabolism, any metabolically related side effects will need to be monitored closely in these patients.

It is clear that available data and evidence supports the PI3K enzymes as attractive targets for future development of drugs. However, as studies of PI3K in platelet function are just recently emerging, more studies are required to determine if PI3K has any clinical potential as anti-thrombotic targets. Despite the promising future of PI3K in a clinical perspective, there are still a lot of unexplored areas in the field.

## Figures and Tables

**Table 1:** Effects on platelet function and thrombus formation of targeting different PI3K isoforms.

PI3K	Species/Model	Platelet/Thrombus Readout				References
		Platelet Function	Thrombus Formation			
			Ex Vivo *	In Vivo **		
				Thrombosis	Bleeding Time	
**Class I PI3K—generates PI(3,4,5)P_3_**						
PI3Kα	Mouse genetic	↓	-	↓	-	[56,57]
	Human—PI3Kα inhibitor	↓	-	n.d.	n.d.	[57,58]
PI3Kβ	Mouse genetic	↓	U/↓	↓	-	[52,55]
	Human—PI3Kβ inhibitor	↓	U	n.d.	↑	[55,58]
PI3Kδ	Mouse genetic	↓	-	n.d.	n.d.	[63]
	Human—PI3Kδ inhibitor	↓	n.d.	n.d.	n.d.	[58]
PI3Kγ	Mouse genetic	↓	U	↓	-	[64,65,66]
**Class II PI3K—generates PI(3)P and PI(3,4)P_2_**						
PI3KC2α	Mouse genetic	-	U	↓	-	[83,84]
	Mouse wild-type with PI3KC2α inhibitor	n.d.	n.d.	↓	-	[87]
	Human—PI3KC2α inhibitor	-	↓	n.d.	n.d.	[87]
	Human genetic—homozygous loss-of-function of PI3KC2α	No thrombotic phenotype reported Developed novel syndrome—dysmorphic facial features, short statures, cataracts, multiple skeletal and neurological abnormalities				[76]
PI3KC2β	Mouse genetic	-	-	-	-	[83]
**Class III PI3K—generates PI(3)P**						
Vps34	Mouse genetic	n.d.	↓	↓	-	[96,97]
	Human—Vps34 inhibitor	↓	↓	n.d.	n.d.	

* Ex vivo thrombus formation assessed by thrombus formation in perfused whole blood over an adhesive protein. U = unstable, n.d. = not determined. ** Thrombosis function assessed by induced vessel injury. Bleeding time reflects haemostasis function. PI3KC2γ is not included in the table as it is not expressed in platelets.
